# Amplicon and Metagenomic Analysis of Middle East Respiratory Syndrome (MERS) Coronavirus and the Microbiome in Patients with Severe MERS

**DOI:** 10.1128/mSphere.00219-21

**Published:** 2021-07-21

**Authors:** Waleed Aljabr, Muhannad Alruwaili, Rebekah Penrice-Randal, Abdulrahman Alrezaihi, Abbie Jasmine Harrison, Yan Ryan, Eleanor Bentley, Benjamin Jones, Bader Y. Alhatlani, Dayel AlShahrani, Zana Mahmood, Natasha Y. Rickett, Bandar Alosaimi, Asif Naeem, Saad Alamri, Hadel Alsran, Maaweya E. Hamed, Xiaofeng Dong, Abdullah M. Assiri, Abdullah R. Alrasheed, Muaawia Hamza, Miles W. Carroll, Matthew Gemmell, Alistair Darby, I’ah Donovan-Banfield, James P. Stewart, David A. Matthews, Andrew D. Davidson, Julian A. Hiscox

**Affiliations:** a Research Center, King Fahad Medical City, Riyadh, Saudi Arabia; b Institute for Infection, Veterinary and Ecological Sciences, University of Liverpool, Liverpool, United Kingdom; c Department of Medical Applied Sciences, Unayzah Community College, Qassim University, Unayzah, Saudi Arabia; d Health Protection Research Unit in Emerging and Zoonotic Infections, Liverpool, United Kingdom; e Department of Microbiology, College of Science, King Saud University, Riyadh, Saudi Arabia; f Assistant Agency of Preventive Health, Ministry of Health, Riyadh, Saudi Arabia; g Public Health England, Salisbury, United Kingdom; h School of Cellular and Molecular Medicine, University of Bristol, Bristol, United Kingdom; i Infectious Diseases Horizontal Technology Centre (ID HTC), Agency for Science, Technology and Research (A*STAR), Singapore; Icahn School of Medicine at Mount Sinai

**Keywords:** MERS-CoV, MinION, metagenomics, sequencing

## Abstract

Middle East respiratory syndrome coronavirus (MERS-CoV) is a zoonotic infection that emerged in the Middle East in 2012. Symptoms range from mild to severe and include both respiratory and gastrointestinal illnesses. The virus is mainly present in camel populations with occasional zoonotic spill over into humans. The severity of infection in humans is influenced by numerous factors, and similar to severe acute respiratory syndrome coronavirus 2 (SARS-CoV-2), underlying health complications can play a major role. Currently, MERS-CoV and SARS-CoV-2 are coincident in the Middle East and thus a rapid way of sequencing MERS-CoV to derive genotype information for molecular epidemiology is needed. Additionally, complicating factors in MERS-CoV infections are coinfections that require clinical management. The ability to rapidly characterize these infections would be advantageous. To rapidly sequence MERS-CoV, an amplicon-based approach was developed and coupled to Oxford Nanopore long read length sequencing. This and a metagenomic approach were evaluated with clinical samples from patients with MERS. The data illustrated that whole-genome or near-whole-genome information on MERS-CoV could be rapidly obtained. This approach provided data on both consensus genomes and the presence of minor variants, including deletion mutants. The metagenomic analysis provided information of the background microbiome. The advantage of this approach is that insertions and deletions can be identified, which are the major drivers of genotype change in coronaviruses.

**IMPORTANCE** Middle East respiratory syndrome coronavirus (MERS-CoV) emerged in late 2012 in Saudi Arabia. The virus is a serious threat to people not only in the Middle East but also in the world and has been detected in over 27 countries. MERS-CoV is spreading in the Middle East and neighboring countries, and approximately 35% of reported patients with this virus have died. This is the most severe coronavirus infection so far described. Saudi Arabia is a destination for many millions of people in the world who visit for religious purposes (Umrah and Hajj), and so it is a very vulnerable area, which imposes unique challenges for effective control of this epidemic. The significance of our study is that clinical samples from patients with MERS were used for rapid in-depth sequencing and metagenomic analysis using long read length sequencing.

## INTRODUCTION

Coronaviruses were once described as the backwater of virology, as they did not cause extensive disease in humans. However, with the emergence of severe acute respiratory syndrome coronavirus (SARS-CoV) in China in 2003, Middle East respiratory syndrome coronavirus (MERS-CoV) in Saudi Arabia in 2012, and now SARS-CoV-2 originating in 2019 in China, this is clearly not the case. These viruses cause respiratory and gastrointestinal illnesses and share similar genome architectures and disease profiles. Patients with existing health care problems, such as underlying cardiovascular disease and genetic factors, may exhibit more severe symptoms and potentially have a fatal outcome (1–4). As such, the presence of other respiratory pathogens may also be of critical importance in the etiology of these infections, and they may also be part of the normal healthy microbiome (5). Severe infection in humans is typified by cytokine storms (6, 7), pneumonia, and kidney failure. MERS-CoV has been identified by the WHO as a prioritized disease and is on their list of pathogens for research and development in emergency contexts. With the advent of SARS-CoV-2, there is an urgent and unmet health care need to develop generic medical countermeasures to combat these infections and mitigate future outbreaks, particularly in contact tracing and understanding transmission dynamics. As a consequence, these viruses cripple national infrastructure, trigger city-wide transport curfews, and disrupt international travel and commerce. Many countries are at risk for both SARS-CoV-2 and MERS-CoV.

There are striking parallels between the emergence of all three of these viruses. For example, one of the major concerns with MERS-CoV is the potential spread of the virus and other pathogens during Hajj (8). This is an annual Islamic pilgrimage to Mecca in Saudi Arabia, involving some 2 million of the world’s population and approximately 0.5 million Saudi residents. There are constant spillover events from camels into humans, and the geographical distribution of MERS-CoV in dromedaries is increasing either through spread and/or increased surveillance. Currently, antiviral therapies and vaccines have compassionate or emergency use for the prevention or treatment of SARS-CoV-2 infection in humans (9–12); however, none exist for MERS-CoV. Several ongoing studies are evaluating medical countermeasures for MERS-coronavirus infection. They have included compounds already in use in the clinic, such as a combination of interferon-α2b and ribavirin (13). A phase 1 DNA vaccine, based on the viral spike glycoprotein, funded by US Department of the Army, has been recently evaluated in a human trial (14) and also in dromedary camels (15). Additionally, a phase 1 clinical trial has started in humans in Saudi Arabia assessing the safety and tolerability of a vaccine based on the ChAdOx1 MERS vaccine (16, 17). The sporadic nature of the outbreaks and the lack of suitable animal models have hindered research.

Given the lack of medical countermeasures, shutting down transmission changes is essential to bringing such outbreaks under control (18), and sequencing the genomes of viruses can aid in this endeavor through molecular epidemiology, as was demonstrated with SARS-CoV in Singapore (19, 20). Sequencing provides information on the rate of evolution of the virus (21), the origin of specific clusters (22), and whether an outbreak is caused by continuous human-to-human transmission (21, 23); or it can be used to investigate zoonotic spillover (24). The use of long read length sequencing centered on the Oxford Nanopore platform has rapidly decreased sequencing turnaround time and can be applied to coronaviruses, for example with SARS-CoV-2 (25, 26).

MERS-CoV is highly transmissible (27), and sporadic outbreaks are ongoing in the Kingdom of Saudi Arabia and surrounding regions. In order to aid in the diagnosis of MERS-CoV by rapidly generating a viral genome sequence and to map the microbiome in the nasopharyngeal sample, an amplicon-based and metagenomic MinION sequencing approach was used. During replication of coronaviruses, with an approximately 30-kb positive-sense RNA genome, a nested set of subgenomic mRNAs are synthesized. Subgenomic mRNAs located toward the 3′ end of the genome are generally more abundant than transcripts nearer the 5′ end (28, 29), and these products can be identified by amplicon sequencing. This study used a mixture of amplicon sequencing and metagenomic approaches to characterize clinical samples from patients with MERS. The amplicon approach was based on generating 30, 15, or 8 overlapping fragments that spanned the genome but depended on the starting material. The rationale for using larger amplicons was to establish a system that could be used to identify deletions or recombination sites in the genome, which are hallmarks of coronavirus RNA synthesis. The data demonstrated that whole-genome or near-whole-genome information on MERS-CoV was rapidly obtained from samples taken from infected patients and sequenced using the amplicon approach. Furthermore, the amplicon-based sequencing approach provided data on both consensus genomes and the presence of minor variants, including deletion mutants. Metagenomic analysis provided information of the background microbiome and how this may be associated with outcome. Real-time analysis of the microbiome in patients and the identification of antibiotic resistance markers may provide better chemotherapeutic approaches to manage coinfections.

## RESULTS AND DISCUSSION

**FIG 1:**
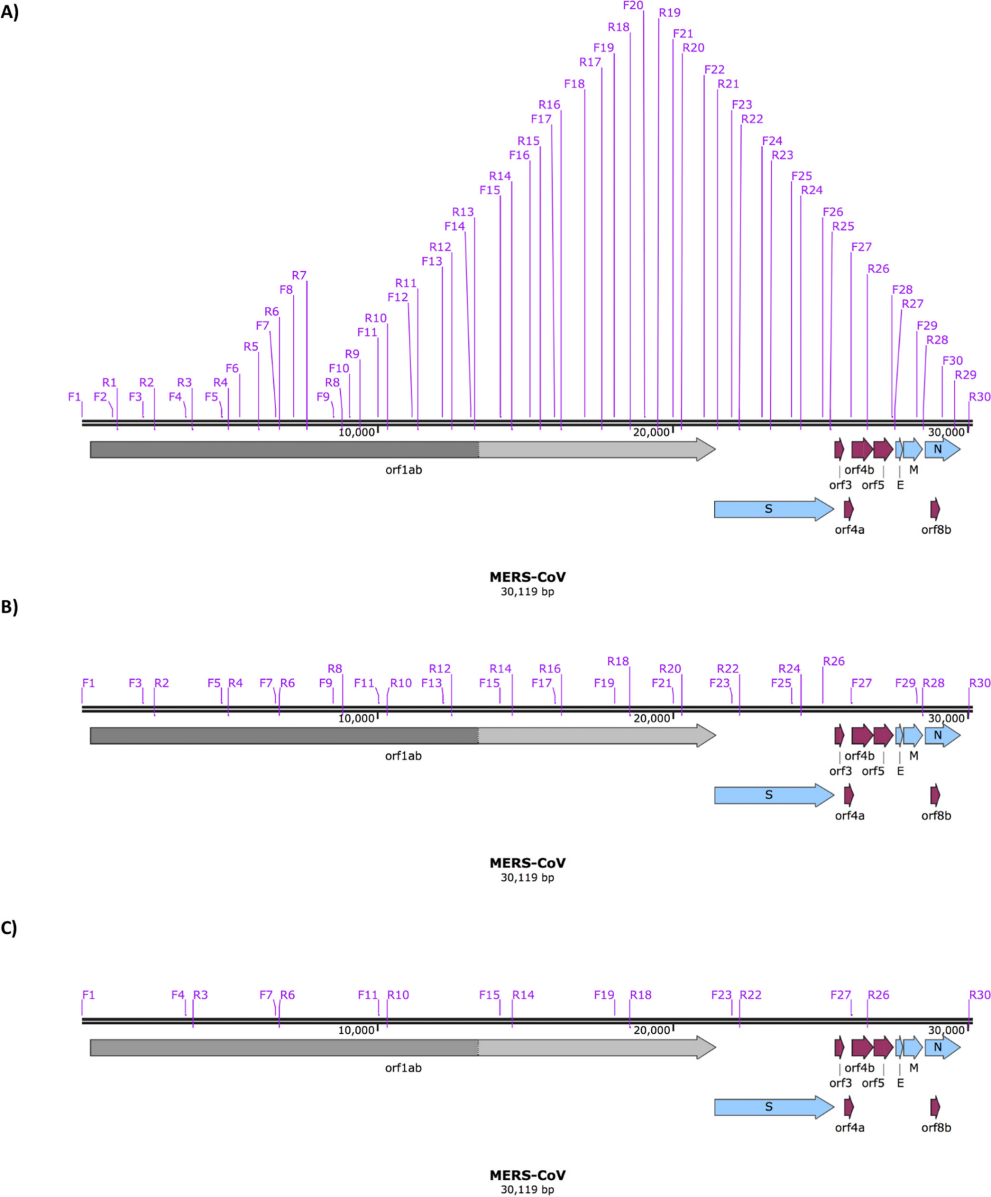
Location of conserved primer pairs (Table 4) on the MERS-CoV genome and position compared with MERS-CoV genes. Primer pairs can be used to generate amplicons of various lengths, including 30, 15, and 8 amplicons as indicated. Appropriate genes are indicated on the MERS-CoV genome.

Single nucleotide polymorphisms, recombination, and resulting deletions (and potentially insertions) may account for the wide genome diversity observed in some strains of coronaviruses (30–32). These recombination events can led to new strains of coronaviruses (33) or potentially affect vaccine strategies (34, 35). In order to identify these genome changes with sequencing for MERS-CoV, rapid long read length approaches offered by Oxford Nanopore were developed. They used longer amplicons selected through appropriate primer pairs located along the MERS-CoV genome (Fig. 1). Note that this approach would also use the nested set of subgenomic mRNAs to capture sequence information.

### Validation of primers and generation of amplicons using total RNA purified from MERS-CoV-infected cells.

**FIG 2:**
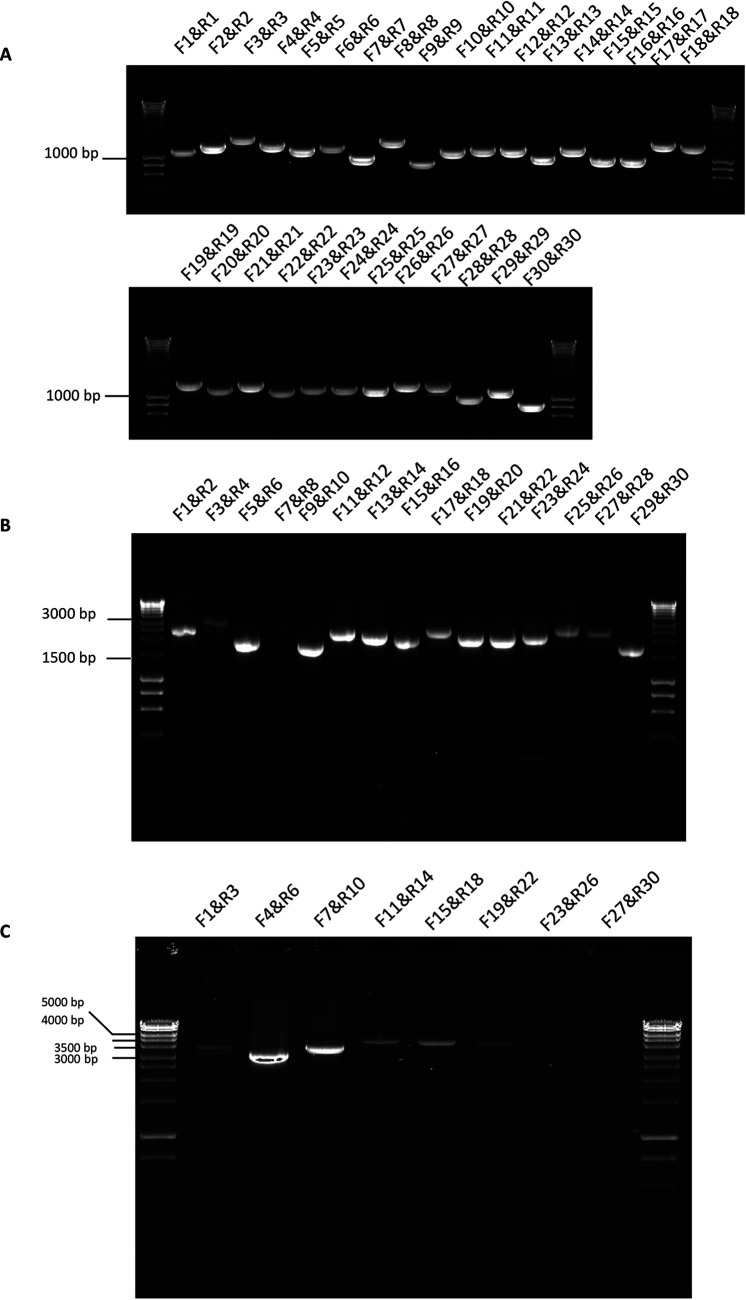
Agarose gel electrophoresis of amplicons generated using 30 (A), 15 (B), and 8 (C) combinations of primers pairs. These primer pairs were used to generate amplicons in combination with reverse transcription of RNA extracted from MERS-CoV-infected cells and are indicated above the appropriate lane on the gel image. Molecular size markers are indicated to the left.

To evaluate the utility of the selected primers for the amplification of viral RNA under controlled conditions, total RNA was purified from MRC-5 cells that had been infected with the EMC strain of MERS-CoV at a multiplicity of infection (MOI) of 5. This RNA was used as a template to prime cDNA synthesis using random hexamers. Amplification conditions are provided in Table 1; the MERS-CoV genome was amplified using either 30 amplicons (Fig. 2A), 15 amplicons (Fig. 2B), or 8 amplicons (Fig. 2C), using the same set of conditions for each set of amplicons (see Table S1 in the supplemental material). The rationale behind using the same amplification conditions across all primer pairs is that amplification would be more efficient if a large-scale sample analysis was required. These data indicated that for the all of the approaches, PCR products were observed that spanned the MERS-CoV genome. However, the 15- and 8-amplicon approach products varied in efficiency.

**TABLE 1:** PCR conditions for 30-, 15-, and 8-amplicon approaches

Step	No. of cycles	Temp (°C)	Time
Initial denaturation	1	98	30 sec
Denaturation	35	98	10 sec
Annealing		66	30 sec
Extension		72	50^*a*^, 90^*b*^, or 180^*c*^ sec
Final extension	1	72	2 min

a For the 30-amplicon approach.

b For the 15-amplicon approach.

c For the 8-amplicon approach.

10.1128/mSphere.00219-21.1 TABLE S1 Details of primer sequences used in this study. The expected product size is given for the 30-amplicon approach (see Fig. 1). Download Table S1, DOCX file, 0.02 MB. Copyright © 2021 Aljabr et al. 2021 Aljabr et al. https://creativecommons.org/licenses/by/4.0/ This content is distributed under the terms of the Creative Commons Attribution 4.0 International license.

### Generation of amplicons from patients infected with MERS-CoV and derivation of consensus genome sequence.

**FIG 3:**
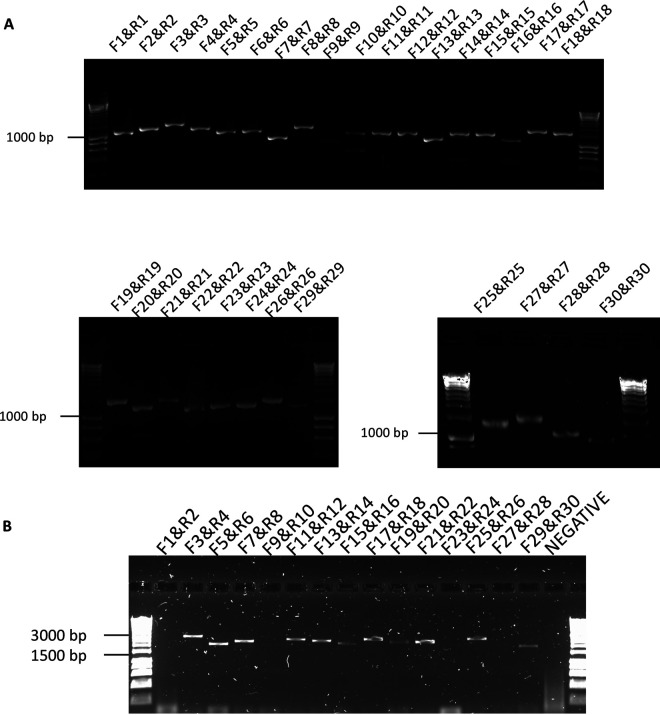
Agarose gel electrophoresis of amplicons generated using 30 (A) and 15 (B) combinations of primers pairs. These primer pairs were used to generate amplicons in combination with reverse transcription of RNA extracted from nasal aspirates taken from patients with MERS. Primer pairs are indicated above the appropriate lane on the gel image. Molecular size markers are indicated to the left.

**FIG 4:**
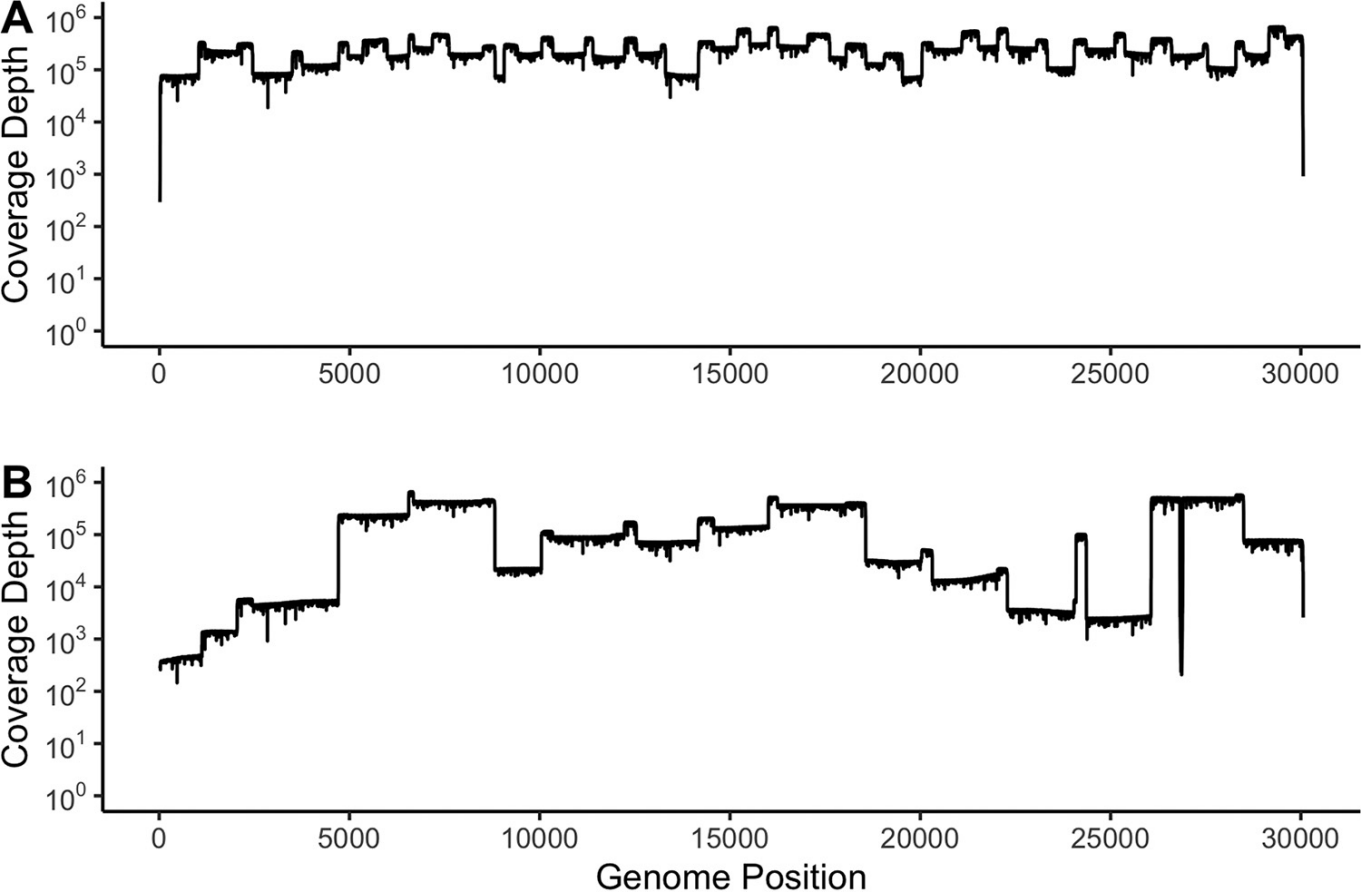
Read depth analysis of 30 (A) and 15 (B) amplicons sequenced on a single flow cell using an Oxford Nanopore MinION device. Coverage of each position on the MERS-CoV genome is indicated on the *y* axis. The nucleotide length of the MERS-CoV genome is indicated by the genome position on the *x* axis. The dashed line represents 20× coverage, indicating that above this line each nucleotide was sequenced at least 20 times.

The 30-, 15-, and 8-amplicon approaches were evaluated on RNA extracted from nasal aspirates taken from patients with MERS. The data indicated that the 30- and 15-amplicon approaches could be used to generate fragments from clinical samples (Fig. 3A and B, respectively). The 8-amplicon approach was not sufficient for obtaining coverage across the MERS-CoV from a clinical sample (data not shown), probably due to the lower quality of RNA than that obtained from cell culture. The PCR products generated in the 30- and 15-amplicon approach (from separate patients) were combined for each patient, barcoded, and sequenced on separate flow cells for each patient. Sequencing reads generated by the MinION were aligned to a reference sequence. The analysis showed that a complete genome sequence could be obtained from the 30-amplicon (Fig. 4A) and 15-amplicon approach (Fig. 4B). A consensus sequence was generated using the ARTIC bioinformatic pipeline. To identify the viral genome sequence that dominated the population, a custom perl script was used to count the number of each nucleotide against the reference sequence (GenBank accession number NC_019843.3). This sequence was used to generate a bespoke consensus genome for an individual patient.

### Analysis of the minor variant population within patients.

**FIG 5:**
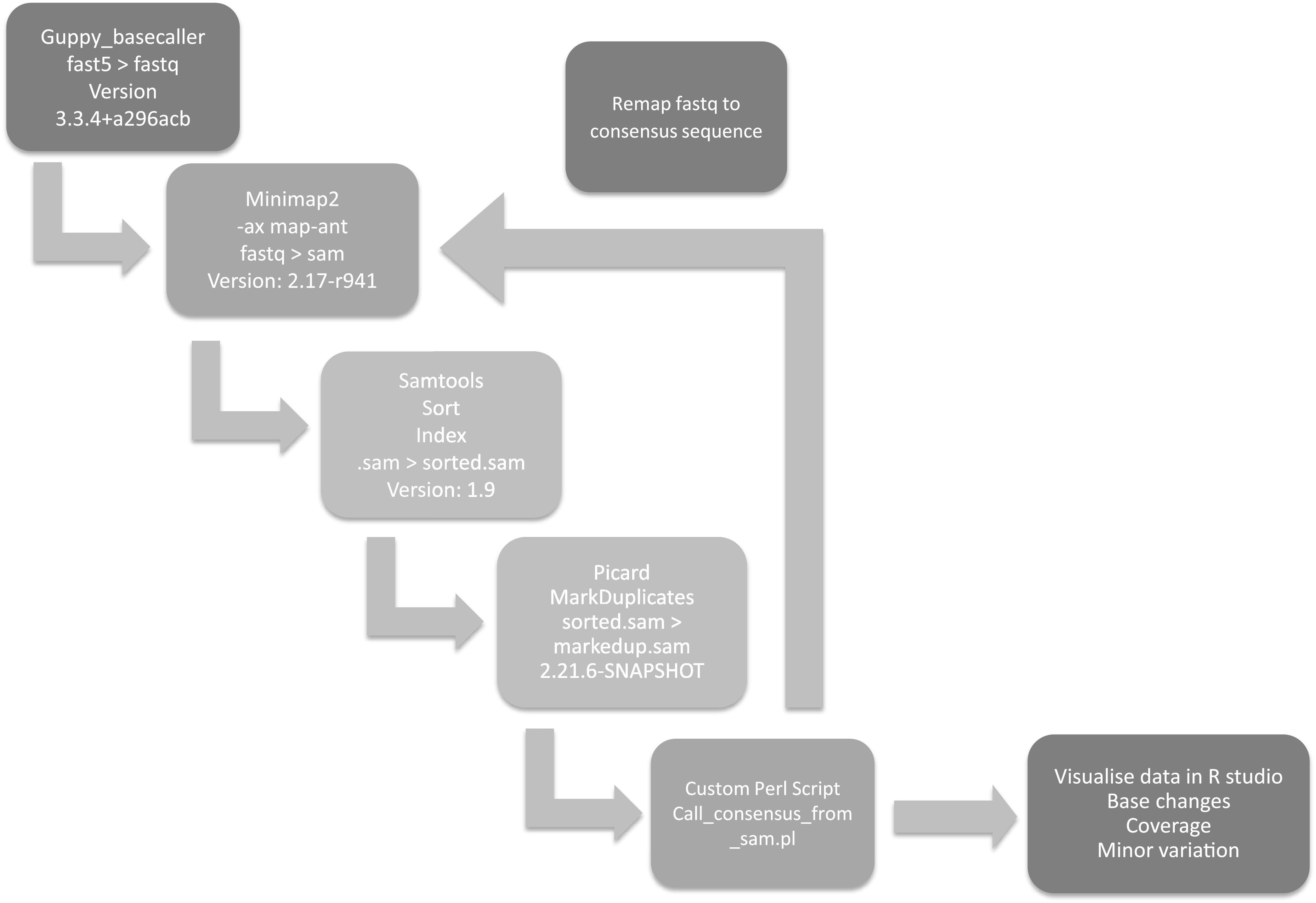
A flow diagram of the bioinformatic pipeline that was used to derive viral genome coverage and minor variation information of viral genomes within patients.

**FIG 6:**
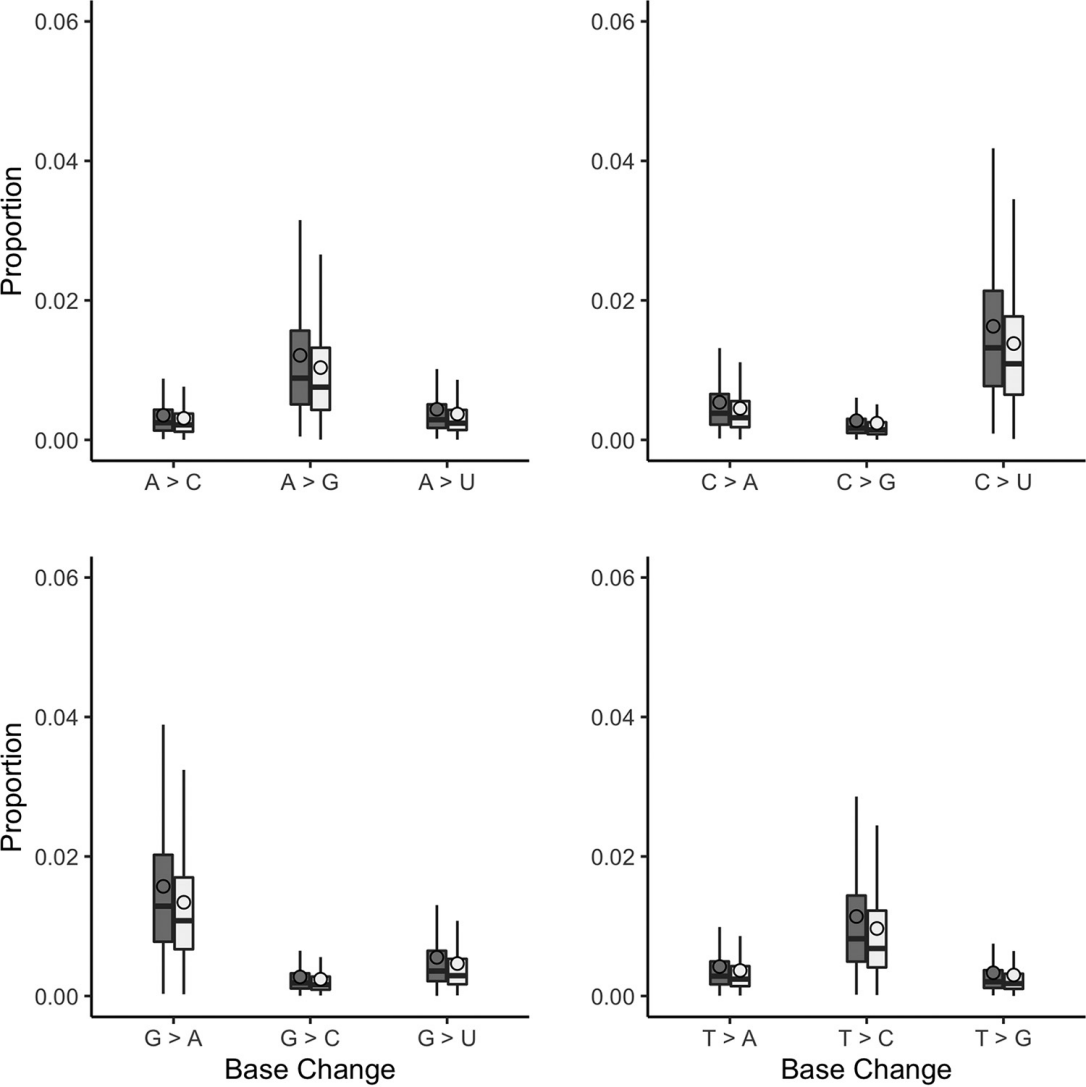
The sequencing reads were mapped to the patient consensus viral genome sequence. The custom script counted the number of each base at each genome position with a quality score of >10. Positions with a depth of <20 were removed from the analysis. This figure shows the proportion of base changes observed compared with the patient’s dominant consensus reference genome. Overall, transitions were observed more frequently than transversions, where C > U is the most observed base change. Patient 10, dark gray; patient 115, light gray; outliers not visualized.

**FIG 7:**
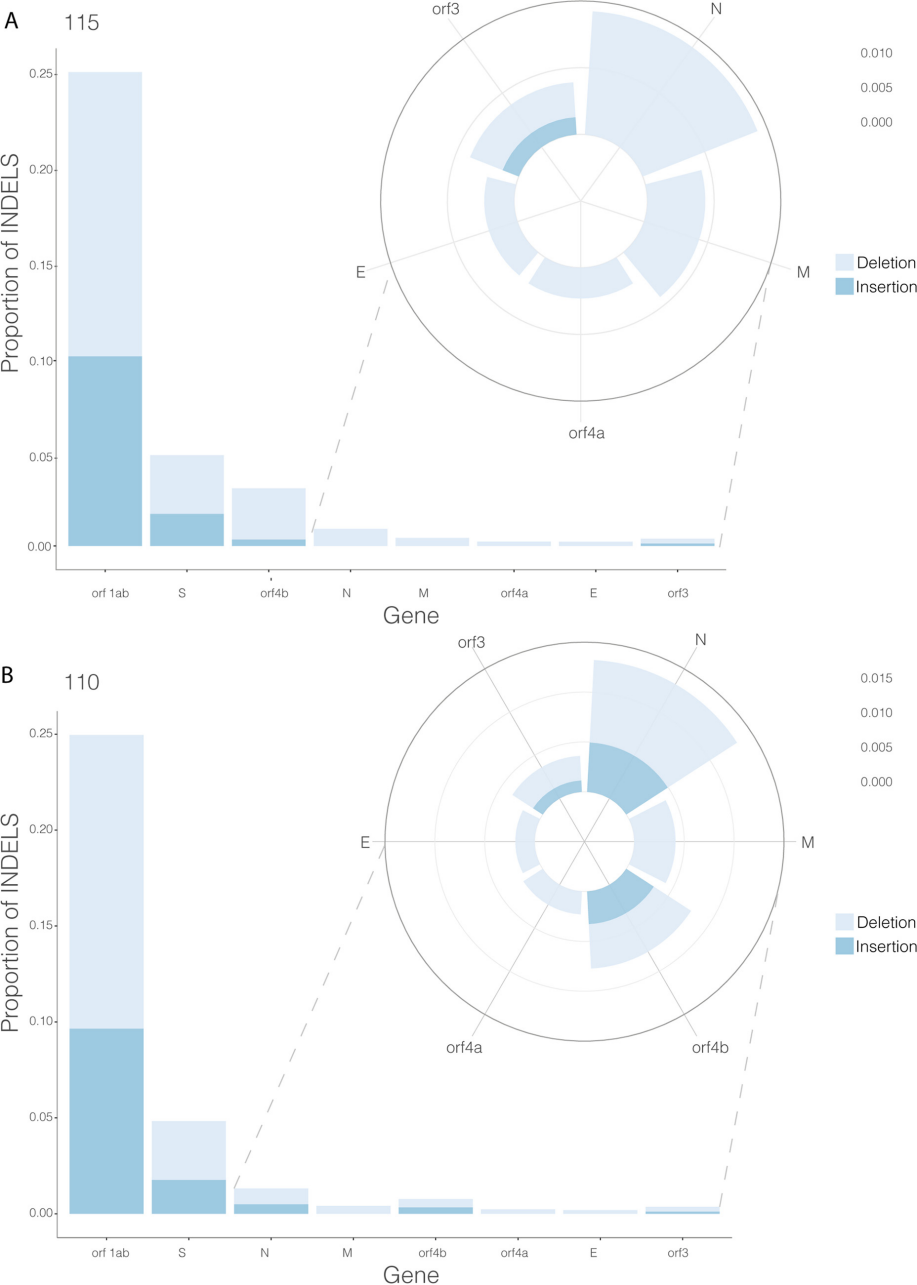
Genome-wide representation of standardized indel polymorphisms for patient (PX) 110 and PX 115. Indels are designated by bars and anchored at the *x* axis, corresponding to each gene. The standardized indel proportion is ranked in each plot. Where the difference in scale hinders visualization, radial subplots are used to provide a view of genes with the lowest proportion of indels.

**FIG 8:**
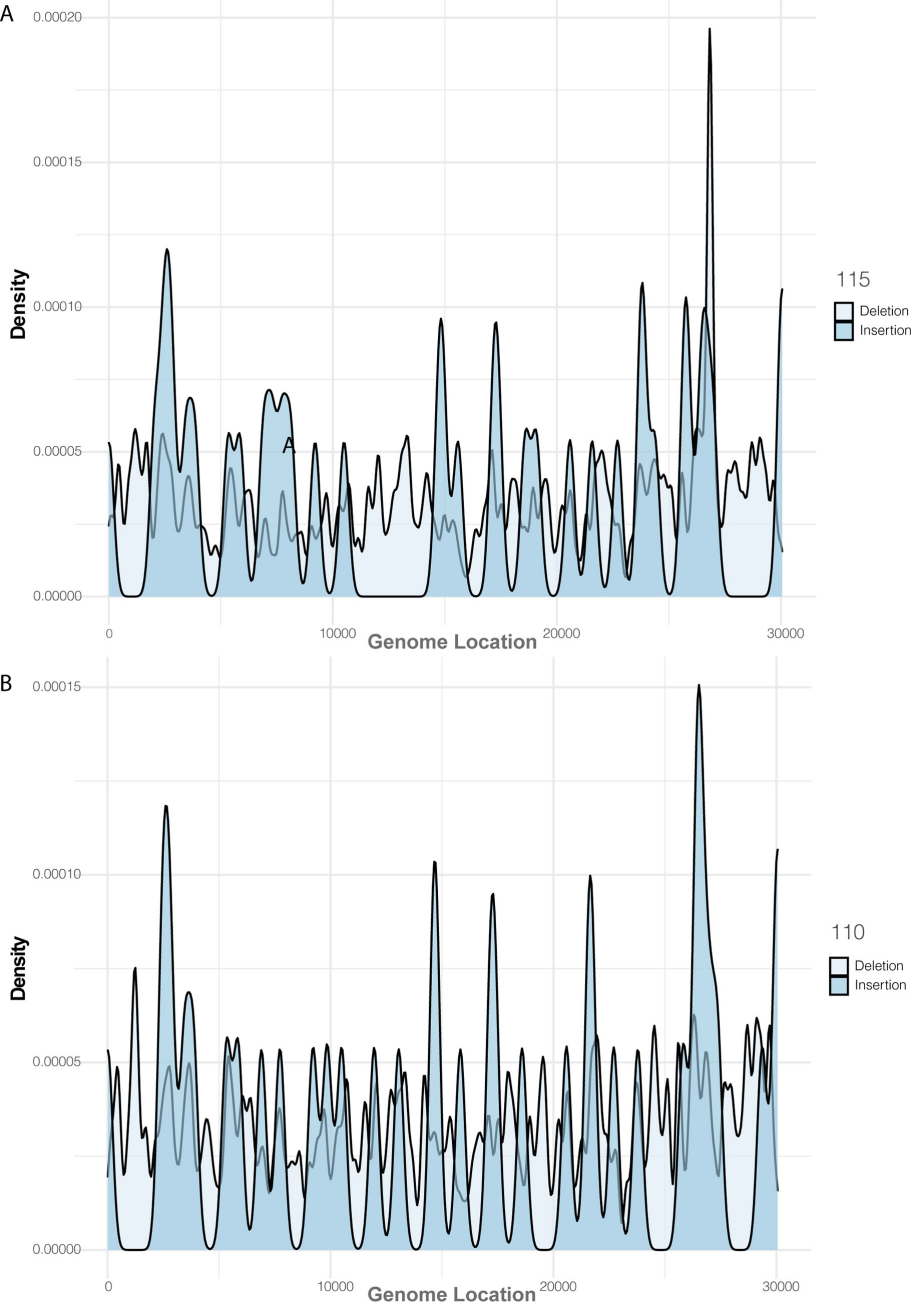
Genome-wide representation of indel polymorphisms, demonstrated by smoothened densities (adjust, 0.05; alpha, 0.7) for PX 110 and PX 115.

The minor variant population in infections has been shown to influence the kinetics of virus replication and be associated with patient outcome (36). Therefore, methodologies were developed that could be used to assess the minor variant frequency within a sample from a patient infected with MERS-CoV. The custom perl script used to call the consensus also revealed the nucleotide depth and the counts of each nucleotide at each position (Fig. 5). The depth was used to normalize the mutation frequency into a proportion instead of a raw count, allowing a comparison of samples of different read depths. Nucleotides that had a count less than 20 were removed from analysis. As proof of principle, this approach was applied to the sequencing data obtained from patients 10 and 115, as these were of higher quality. Patient 10 appeared to have more base changes than patient 115 (Fig. 6). Transitions (A > G, G > A, C > U, and U > C) were more frequently observed, and C > U seemed more prominent than other mutations. This finding is consistent with RNA editing by APOBEC. APOBEC3 family members have been shown to be involved in restricting the growth of human coronavirus NL63 (HCoV-NL63) (37) and identified as potential drivers of C-to-U transitions in SARS-CoV-2 (38). We note that assessing minor variant populations using data from MinION sequencing may be problematic due to the higher error rate in sequencing than for example Illumina-based approaches (39). However, with sufficient read depth in a sample, an overview of the minor variant frequency/population can still be obtained. Adjusted gene size was calculated by dividing each MERS gene by the length of the total viral genome (30,108 bp). Global indel polymorphisms were grouped by category and mapped to their corresponding location within the reference sequence (Fig. 7). The mean proportion of each observation (against depth) was multiplied by the adjusted gene size to produce a standardized indel proportion for each gene (Fig. 8).

### Identification and analysis of deletions in the viral genome in samples from patients.

Deletions within the genome of MERS-CoV were identified in the sequencing data from patients 10 and 115 (Table 2). Patient 10 had five deletions in Orf1ab and a deletion spanning N. Patient 115 was sequenced with the 15-amplicon approach and therefore generated amplicons over 2 kb in length. A deletion of 77 bases that spanned the *orf4b* and *orf5* genes was identified in this patient. Deletions in this region may have implications on virus pathogenesis, ORF4A is able to inhibit early antiviral responses (interferon α/β [IFN α/β]) in the host (40), and likewise ORF5 may reduce inflammatory responses (41). This finding was consistent in both bioinformatic pipelines that were used. The presence of these deletions does not rule out that at the minor variant level full intact gene were present. However, the data do illustrate the plasticity of the MERS-CoV genome.

**TABLE 2:** Analysis of deletions present in the MERS-CoV genome from patients 115 and 10

Deletion information							Affected gene information							
Patient no.	Start position (bp)	End position (bp)	No. of supporting reads	Quality score^*a*^	SD of span	SD of position	Gene start	Gene end	Gene name	Overlap (bp)	Gene start	Gene end	Gene name	Overlap (bp)
115	26820	26897	97	99	1.96	1.83	26093	26833	*orf4b*	13	26840	27514	*orf5*	57
10	1315	2149	6	7	0.41	0.2	279	21514	*orf1ab*	834				
	3576	4551	10	12	3.46	57.21	279	21514	*orf1ab*	975				
	7611	8507	31	38	0.62	0.23	279	21514	*orf1ab*	896				
	8818	9056	22	27	2.63	3.02	279	21514	*orf1ab*	238				
	9407	10044	14	17	0.27	0.4	279	21514	*orf1ab*	637				
	28514	29110	40	49	56.94	15.59	28566	29807	N	544				

a Takes into consideration the mapping quality scores, where a value greater than 10 has higher confidence.

### Identification of bacterial and viral sequences in samples from patients and antibiotic resistance markers.

**FIG 9:**
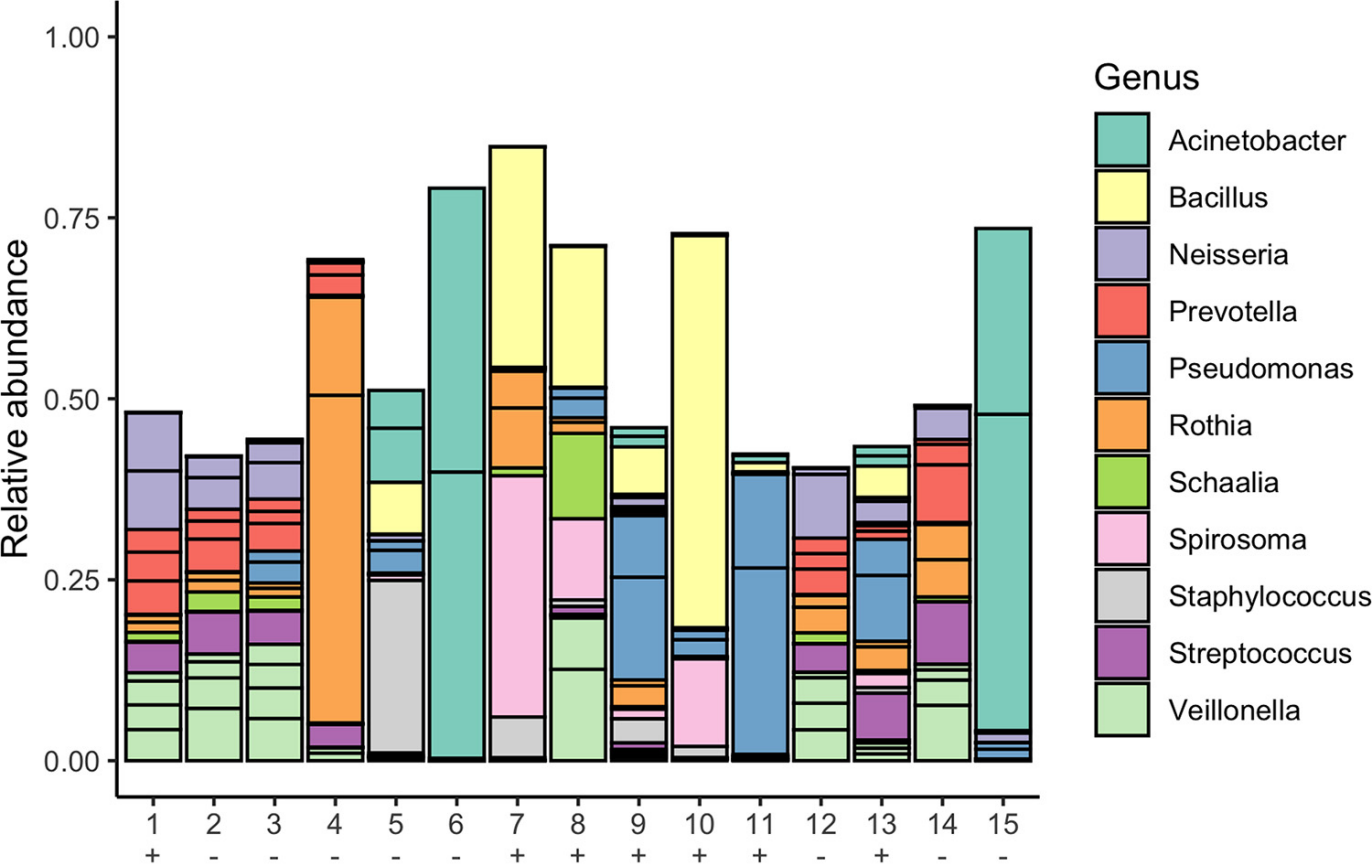
The top 20 species categorized into genus from 15 patients with severe MERS infection. Human reads were removed from sequencing libraries, and viral and bacterial transcripts were identified using Kraken2. Kraken2 outputs were converted into biom format before importing into R with Phyloseq. The relative abundance of each species is plotted for each patient. +, MERS-CoV reads detected; −, MERS-CoV reads not detected.

The presence of other infections in patients with MERS may also require clinical management and influence the outcome. A metagenomic approach was used to identify other microorganisms present in the clinical samples from patients with MERS. Species such as Acinetobacter baumannii, Pseudomonas aeruginosa, and Streptococcus pneumoniae were identified, of which all can be associated with bacterial pneumonia (Fig. 9). Although the detection of RNA does not always infer active infection, identification is based on the active bacterial transcriptome.

Patients with Acinetobacter transcripts were associated with a fatal outcome of the disease (Fig. 9). Acinetobacter infections are often exclusive to health care settings, especially in patients who have received ventilation support. Acinetobacter species are considered a serious multidrug-resistant pathogen and are encompassed in the ESKAPE acronym, referring to Enterococcus faecium, Staphylococcus aureus, Klebsiella pneumoniae, Acinetobacter baumannii, Pseudomonas aeruginosa and Enterobacter species (42). Evidence for antibiotic-resistant genes was identified in patient 6 within Klebsiella pneumoniae (TEM-4) and Acinetobacter baumannii species (*LpxC*, *adeI*, *adeJ*, *mexT*, *adeN*, *adeK*, and ADC-2) (Table 3). This patient died from a severe MERS infection. Several groups have suggested that the widespread use of antibiotics as part of the management of patients with COVID-19 may effect antimicrobial resistance (AMR) (43). Metagenomic analysis of samples from patients with severe coronavirus infection, as illustrated in this study, can be used to track these markers. *Human alphaherpesvirus 1* transcripts were identified in patient 5 through this approach. Ongoing research throughout the SARS-CoV-2 pandemic suggests that coronaviruses may be able to reactivate herpes simplex virus and cytomegalovirus during severe disease (44).

**TABLE 3:** Antimicrobial resistant genes identified in bacteria from patients with MERS-CoV infection^*a*^

Patient no.	Gene	No. of alignments	Avg accuracy (%)	Taxon	CARD model
4	Escherichia coli 16S rRNA mutation in the *rrsB* gene conferring resistance to tetracycline	357	91.90	Escherichia coli K-12	rRNA mutation model
	Escherichia coli 16S rRNA mutation in the *rrsB* gene conferring resistance to neomycin	381	91.80	Escherichia coli K-12	rRNA mutation model
	Escherichia coli 16S rRNA mutation in the *rrnB* gene conferring resistance to streptomycin	365	91.80	Escherichia coli K-12	rRNA mutation model
	Escherichia coli 16S rRNA mutation in the *rrnB* gene conferring resistance to spectinomycin	380	91.60	Escherichia coli K-12	rRNA mutation model
	Escherichia coli 16S rRNA mutation in the *rrsB* gene conferring resistance to gentamicin C	332	91.60	Escherichia coli K-12	rRNA mutation model
	Escherichia coli 16S rRNA mutation in the *rrsB* gene conferring resistance to streptomycin	378	91.60	Escherichia coli K-12	rRNA mutation model
	Escherichia coli 16S rRNA mutation in the *rrnB* gene conferring resistance to tetracycline	387	91.50	Escherichia coli K-12	rRNA mutation model
	Escherichia coli 16S rRNA mutation in the *rrsB* gene conferring resistance to spectinomycin	339	91.5	Escherichia coli K-12	rRNA mutation model
	Escherichia coli 16S rRNA mutation in the *rrsB* gene conferring resistance to tobramycin	407	91.50	Escherichia coli K-12	rRNA mutation model
	Escherichia coli 16S rRNA mutation conferring resistance to edeine	331	91.40	Escherichia coli K-12	rRNA mutation model
	Escherichia coli 16S rRNA mutation in the *rrsB* gene conferring resistance to G418	340	91.40	Escherichia coli K-12	rRNA mutation model
	Escherichia coli 16S rRNA mutation in the *rrsB* gene conferring resistance to paromomycin	329	91.00	Escherichia coli K-12	rRNA mutation model
	Escherichia coli 16S rRNA mutation in the *rrsB* gene conferring resistance to kanamycin A	356	90.80	Escherichia coli K-12	rRNA mutation model
	Escherichia coli 16S rRNA mutation in the *rrsH* gene conferring resistance to spectinomycin	389	90.60	Escherichia coli K-12	rRNA mutation model
	Escherichia coli 16S rRNA mutation in the *rrsC* gene conferring resistance to kasugamycin	420	90.50	Escherichia coli K-12	rRNA mutation model
6	TEM-4	29	95.00	Klebsiella pneumoniae	Protein homolog model
	*LpxC*	80	94.30	Acinetobacter baumannii	Protein variant model
	*adeI*	30	93.70	Acinetobacter baumannii AB0057	Protein homolog model
	*adeJ*	36	93.40	Acinetobacter baumannii	Protein homolog model
	*mexT*	29	93.30	Acinetobacter baumannii SDF	Protein homolog model
	*adeN*	18	92.70	Acinetobacter baumannii	Protein homolog model
	*adeK*	21	92.60	Acinetobacter baumannii	Protein homolog model
	ADC-2	18	92.10	Proteobacteria	Protein homolog model
15	*msr*(E)	5	93.80	Escherichia coli	Protein homolog model

a The sequence-independent, single-primer amplification (SISPA) method was used to identify viral and bacterial transcripts within clinical samples. Fastq files were uploaded to the Oxford Nanopore Technology (ONT) cloud-based pipeline EPI2ME (Fastq antimicrobial resistance, WIMP [rev. 3.4.0], and ARMA CARD [rev. 1.1.6]) workflow to retrieve taxonomy classification and antimicrobial resistance information from MERS-CoV clinical samples. Antimicrobial resistant gene candidates with more than 5 alignments and an average accuracy of more than 90% are presented.

**FIG 10:**
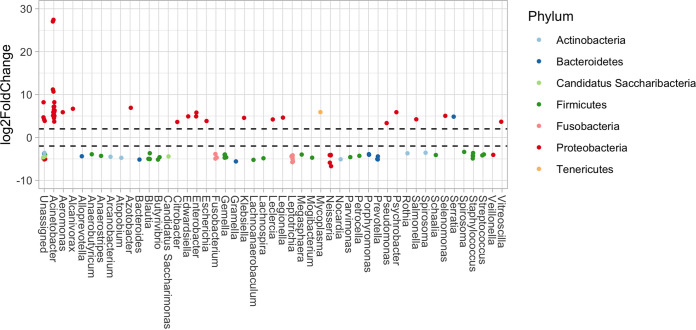
Increased and decreased abundance of bacteria in fatal cases of MERS-CoV infections compared with nonfatal cases derived from transcript abundance data. A Phyloseq object was converted into a DESeq2 object, a contrast between fatal and nonfatal outcome was used to calculate log_2_ fold change, and values with a false discovery rate (FDR) of <0.01 were plotted. The *x* axis represents the genus of identified transcripts, unassigned refers to transcripts that could not be assigned at the genus level, and color illustrates the phyla.

The abundance of bacteria was compared between fatal cases and nonfatal cases using DESeq2 (Fig. 10). In general, transcripts from bacteria in the *Proteobacteria* phyla were higher in abundance in the fatal cases than those in nonfatal cases, including species from the Acinetobacter and Klebsiella genera. Previous studies have suggested interactions between viral infections and bacterial communities. An increase in the abundance of *Proteobacteria* was observed following an experimental rhinovirus infection in chronic obstructive pulmonary disease (COPD) patients (45). Patients with 2009 influenza A H1N1 infections and pneumonia were found to have an expansion of *Proteobacteria* compared with nonviral pneumonias (46–48).

The data indicated that an amplicon-based approach could be used to rapidly sequence MERS-CoV from clinical samples and provide information on genetic diversity and insertions/deletions. This approach was complemented with a metagenomic approach that was able to resolve the microbiome present in the clinical samples. This analysis identified bacteria associated with ventilation and also antibiotic resistance markers. Overall, the research demonstrates the utility of rapid long read length sequencing for characterizing MERS-CoV infection in samples from humans with MERS.

## MATERIALS AND METHODS

### Ethics statement.

Ethical approval was obtained from the Institutional Review Board no. 18-102, King Fahad Medical City, Riyadh, Saudi Arabia. Samples for diagnostic purposes from nasopharyngeal aspirates (NPAs), oropharyngeal swabs, tracheal aspirates, and throat swabs were used for this study.

### Sample collection and processing.

NPAs, oropharyngeal swabs, tracheal aspirates, and throat swabs were collected from MERS-CoV-positive patients (45 to 74 years old) admitted to different hospitals within Saudi Arabia. MERS-CoV diagnosis was confirmed by reverse transcriptase PCR (RT-PCR) (bioMérieux Diagnostics). For this study, the NPAs with a confirmed MERS-CoV diagnosis from the Ministry of Health (MOH) Saudi Arabia, had no identifying information. The sampling NPAs, oropharyngeal swabs, tracheal aspirates, and throat swabs was carried out as per the MOH’s guidelines. Samples were stored at −80°C until used. RNA from the NPAs was extracted using an EZ1 virus minikit v2 (955134; Qiagen). The RNA concentration was measured by the Qubit RNA broad-range (BR) assay (32852; Qiagen). Information on samples from patients used in this study is included in Table 4. This information includes sex, age, hospital, specimen type, threshold cycle (*C_T_*) value of the E gene and ORF1AB, comorbidities, outcome, and whether the patient was in an intensive care unit.

**TABLE 4:** Characteristics of patients included in this study

Patient	Sex	Age	Hospital	Specimen type	MERS-CoV *C_T_* value for:		Comorbidities	Outcome	ICU status
					E gene	*orf1ab*			
1	Male	64	Medinah	Combined nasopharyngeal and oropharyngeal swab	30.84	30.75	Diabetes, hypertension	Nonfatal	
2	Male	64	Medinah	Combined nasopharyngeal and oropharyngeal swab	33.75	33.47	Diabetes, hypertension	Nonfatal	
3	Male	80	Medinah	Combined nasopharyngeal and oropharyngeal swab	30.76	30.96		Fatal	
4	Male	60	Jeddah	Nasopharyngeal swab	21.05	22.73	Diabetes, hypertension, heart disease	Fatal	Yes
5	Male	51	Jeddah	Tracheal aspirate	27.75	29.68		Fatal	Yes
6	Male	82	Jeddah	Nasopharyngeal swab	29.18	29.54	Diabetes	Fatal	Yes
7	Female	64	Jeddah	Nasopharyngeal swab	20.77	20.89		Nonfatal	
8	Male	74	Dammam	Combined nasopharyngeal and oropharyngeal swab	23.49	24.81		Fatal	Yes
9	Female	74	Dammam	Nasopharyngeal swab	24	24	Diabetes, hypertension	Nonfatal	Yes
10	Male	60	Dammam	Combined nasopharyngeal and oropharyngeal swab	19.11	21.26		Fatal	Yes
11	Female	45	Medinah	Combined nasopharyngeal and oropharyngeal swab	27.29	29.77			
12	Male	61	Medinah	Combined nasopharyngeal and oropharyngeal swab	29.78	31.77	Diabetes	Nonfatal	No
13	Male	51	Medinah	Combined nasopharyngeal and oropharyngeal swab	29.27	31.78		Nonfatal	No
14	Male	60	Medinah	Combined nasopharyngeal and oropharyngeal swab	30.02	30.91		Nonfatal	
15	Male	67	Dammam	Throat swab	30	31		Fatal	Yes
115	Male	46	Riyadh	Nasopharyngeal swab	23.37	23.98	Diabetes, hypertension	Nonfatal	Yes

### Virus stock generation and infection.

To prepare RNA from virus-infected cells as a control for amplification, the MRC-5 cell line was infected with MERS-CoV (Erasmus strain) at an MOI of 5. Total RNA was purified using the TRIzol method.

### DNase Treatment of RNA.

RNA was extracted from respiratory samples taken from 15 patients with severe MERS, including combined nasal and oropharyngeal swabs (*n* = 9), a throat swab (*n* = 1), nasal swabs (*n* = 4), and tracheal aspirate (*n* = 1). Metagenomic approaches have been used to identify the microbiome in samples from patients and correlate them with patient outcome (49). Sequence-independent, single-primer amplification (SISPA) was used to identify bacterial and viral transcripts present within the clinical samples. These transcripts were assessed using the real-time analysis pipelines EPI2ME with AMR or Kraken and Phyloseq packages. RNA samples were treated with Turbo DNase (Invitrogen, Vilnius, Lithuania) by adding 1 μl of Turbo DNase with 5-μl 10× buffer in a 56-μl reaction. The reaction mixture was incubated at 37°C for 30 minutes. A total of 5 μl of inactivating agent was added and the mixture incubated at room temperature for 2 minutes. The reaction was centrifuged at 10,000 × *g* for 90 s, and the RNA supernatant was transferred into a new tube.

### Primer design and synthesis.

Primers for the generation of overlapping amplicons were designed using the Primer3Plus platform and rechecked again by Primer BLAST (NCBI) to avoid primers with a high self-complementary score. Primers were synthesized by Eurofins Genomics (the sequence of the primers is shown Table S1). The stock concentration was 100 μM, and primers were diluted in DNase/RNase-free H_2_O to make a 10 μM working concentration. Twenty MERS-CoV genome sequences were aligned with a reference sequence (NC_019843.3; the Erasmus Medical Centre [EMC] sequence). These sequences represented viruses collected in regions of Saudi Arabia and countries that reported cases, including South Korea. Primer binding sites were chosen from conserved regions after alignment so that a minimum of roughly 1,000-bp sequential amplicons would be generated with an approximately 200-bp overlapping region at each terminus. This process resulted in the selection of 30 sets of primer pairs (Table S1) that could be used to walk across the MERS-CoV genome with overlap between each generated amplicon. Alternatively, primer pairs could be selected that allowed the generation of larger amplicons that spanned more of the genome in contiguous reads (Fig. 1). To evaluate the utility of the selected primers for the amplification of viral RNA under controlled conditions, RNA was purified from MRC-5 cells that had been infected with the EMC strain of MERS-CoV at an MOI of 5. Infection was carried out under CL3+ conditions and total RNA purified from these infected MRC-5 cells at 16 h postinfection. This RNA was used as a template to prime cDNA synthesis using random hexamers. Primer pairs (Table S1) were tested by gradient PCR to determine the optimum annealing temperature (data not shown).

### cDNA synthesis and PCR.

Superscript IV reverse transcriptase (18090010; ThermoFisher) and random hexamers (2.5 μM) were used to generate cDNA templates from RNA. cDNA was amplified using a Q5 high-fidelity DNA polymerase (M0491; New England BioLabs [NEB]).

### Amplicon analysis and sequencing.

PCR products were run on a 1% agarose gel in Tris-acetate-EDTA (TAE) buffer to confirm the presence of amplicons. The amplicons generated from an individual patient were pooled and cleaned using AMPure XP beads (A63882; Beckman Coulter) following the manufacturer’s instructions before preparing the sequencing library with the ligation sequencing kit (SQK-LSK109; Oxford Nanopore Technologies). The sequencing library was added to a flow cell connected to a MinIT device, and sequencing was initiated through MinKNOW. An initial rapid base-calling setting was used.

### SISPA.

RNA was reverse transcribed with SuperScript IV reverse transcriptase (Invitrogen) using Sol-PrimerA (5′-GTTTCCCACTGGAGGATA-N9-3′), followed by second-strand DNA synthesis with Klenow (NEB). Reaction conditions for round A were as follows: 1 μl of Sol-PrimerA (40 pmol/μl) was added to 4 μl of sample RNA, which was heated at 65°C for 5 min and then cooled on ice for 5 min. Then, 7 μl of SuperScript master mix (4 μl 5× SuperScript IV buffer, 1 μl 100 mM dithiothreitol [DTT], and 1 μl RNaseOUT [Invitrogen]) and 1 μl SuperScript IV reverse transcriptase (200 U/μl) were added and incubated at 23°C for 10 minutes and then at 50°C for 10 minutes. The reaction was inactivated by incubating at 80°C for 10 minutes. For second-strand synthesis, Klenow fragment (NEB) was used. The cDNA from each reaction mixture was purified using a 1:1 ratio of AMPure XP beads (Beckman Coulter, USA). A total of 5 ml cDNA was amplified with Q5 high-fidelity DNA polymerase (NEB) according to manufacturer’s instructions with Primer Sol-B. Cycling conditions were as follows: 98°C for 30 s; followed by 30 cycles of 98°C for 10 s, 55°C for 30 s, and 72°C for 1 min; with a final extension at 72°C for 10 min. All amplified cDNA was purified using a 1:1 ratio of AMPure XP beads (Beckman Coulter, USA) and resuspended in 25 ml. Purified cDNA was quantified using the Qubit double-stranded DNA (dsDNA) high-sensitivity (HS) assay (Q32851; Invitrogen) according to the manufacturer’s instructions.

### Bioinformatics.

Fast5 files were base called again using Guppy (v.3.5.2) to mitigate for the lower accuracy associated with the rapid base calling setting initially used on the MinIT device. To investigate deletions in the MERS-CoV genome, library reads were filtered by the expected amplicon size and then aligned to the NCBI MERS reference NC_019843.3 using minimap2 (v.2.17-r941) as per the artic pipeline, and SVIM (v.1.4.2) was used to identify deletions (26).

In addition, SAMtools (v.1.10) was used to sort and index the alignment files, picard MarkDuplicates (v.2.23.4) was used to remove amplification duplicates, and then a custom perl script was used count the nucleotides at each position on the reference genome, when the mapping quality was above 10. This step ensures that every nucleotide on the derived consensus genome has been sequenced at least 10 times. Data were visualized using R studio using ggplot2. To consider the minor variation, the nucleotide depth at a single position with less than 20 counts was not taken forward into analysis to mitigate random sequencing errors.

For rapid assessment of the metatranscriptome approach, Fastq files were uploaded to the Oxford Nanopore Technology (ONT) cloud-based pipeline EPI2ME (Fastq antimicrobial resistance; WIMP [rev. 3.4.0], and ARMA CARD (rev. 1.1.6)) workflow to retrieve taxonomy classification and antimicrobial resistance information from MERS-CoV clinical samples.

In addition, metatranscriptomic reads were assessed with Kraken2 (v.2.2.1). Host removal was carried out to remove human reads. The human genome assembly GRCh38.p13 was used as a reference. The human genome assembly was indexed with minimap2 using the parameter “-ax map-ont” (50). The quality-controlled Oxford Nanopore fastq reads were mapped to the indexed human genome assembly with minimap2 using default parameters. The resulting SAM file was sorted and converted to bam with the command “samtools sort” (51). A fastq file with unmapped reads was produced with the “samtools fastq” command with the parameter “-f 4” (51).

The quality-controlled, host-removed reads were classified using Kraken2 (52). The standard Kraken2 output and sample report output files were produced with the options “–output” and “–report,” respectively. The database used during Kraken2 classification consisted of the “bacteria” and “viral” kraken2 reference libraries (built 23 April 2020). Visualisation of the Kraken2 output was carried out with Pavian, utilizing the Kraken2 report files (53). Kraken-biom (https://github.com/smdabdoub/kraken-biom) was used to generate a biom table which was then read into R studio with Phyloseq to plot alpha diversity and abundance of species identified in the clinical samples at the genus level (54). DESeq2 was used to compare the difference in abundance of species between fatal (*n* = 7) and nonfatal (*n* = 7) MERS-CoV infections (55).
