# Supplementary material for: Amplicon and Metagenomic Analysis of Middle East Respiratory Syndrome (MERS) Coronavirus and the Microbiome in Patients with Severe MERS
Source: mSphere. 2021 Jul 21;6(4):e00219-21. doi: 10.1128/mSphere.00219-21 (PMC8386452; doi:10.1128/mSphere.00219-21)
Supplement: TABLE S1 [file msphere.00219-21-st001.docx]

| Primer name | Primer sequence (5′-3′) | Expected product size |
| --- | --- | --- |
| MERS-F1  MERS-R1 | TTGGCTATCTCACTTCCCCT  GAAACGCCACTCCACTTGTT | 1179 |
| MERS-F2  MERS-R2 | GGTTTGGCATGTTGAGCGTA  CAGCCCAGGAGACCTTTGTA | 1418 |
| MERS-F3  MERS-R3 | TCACAGCGTGTTGTACAGAG  TACCACCGCCATGCTTAAGA | 1694 |
| MERS-F4  MERS-R4 | CTCAGACTATCCAGCCCGAG  GTAGGATCAACGGGGCCATA | 1465 |
| MERS-F5  MERS-R5 | GCACGACACAGCAGTTAACA  AACAACACATCGCCGTCTTC | 1273 |
| MERS-F6  MERS-R6 | TTACGTGGGTGTGCAAACTG  CTGTGGTGCCGAGTTTTGAA | 1334 |
| MERS-F7  MERS-R7 | AAGAGTGTAGTGCGGCATCT  CGGTAGTGAGGTCATTTGCG | 1054 |
| MERS-F8  MERS-R8 | ACATGCTCTATACCTCGGCC  CACCAGCAACTCCAGCAATT | 1650 |
| MERS-F9  MERS-R9 | TGGTTTAATGCGTTGCGTGA  CAAAGTCAGGAACGCACACA | 1650 |
| MERS-F10  MERS-R10 | CGTACAGTCAGATGAGGCCT  AAAGTGTAGGCTGGAGTGCT | 900 |
| MERS-F11  MERS-R11 | CCAGTGGAGATGTTGAGGCT  CCACATTACACCACTGCACA | 1346 |
| MERS-F12  MERS-R12 | GTCTACGAGCCCACTACTCC  GTAATGTCCCACAAAGCCCC | 1349 |
| MERS-F13  MERS-R13 | GCAGTGGCCCGTAAGTTAGA  ACTGAGCAGGGATTTGGACA | 1072 |
| MERS-F14  MERS-R14 | ATGGTGGAGCTTCAGTGTGT  GCGTTAGAGGAGGCAATGTG | 1380 |
| MERS-F15  MERS-R15 | CCGCTGAGACACATAGGGAT  AGTTGTCGCCTGCAAAATGT | 1349 |
| Primer name | Primer sequence (5′-3′) | Expected product size |
| MERS-F16  MERS-R16 | CAAAGTTCTACGGTGGCTGG  AGGGAAGTCTGTGAATGGCA | 1063 |
| MERS-F17  MERS-R17 | ACCCTCTCACAAAGCATGAA  GGCCTGAAAGCTCCTTCTTG | 1605 |
| MERS-F18  MERS-R18 | CACTGTTCAGGGACCACCTG  TGAGCCCAACAAACAAACGT | 1524 |
| MERS-F19  MERS-R19 | CTGGCCTCTCACCTGCTTAT  TAACACCAAAGGCGGAAACC | 1477 |
| MERS-F20  MERS-R20 | TTTGATGCACAGCCCTTGAC  GCAAGTGAAGACCGCCTAAC | 1264 |
| MERS-F21  MERS-R21 | CAATGGTGCTATCATCCGTG  ATCTGGCCCTACATCAACGT | 1487 |
| MERS-F22  MERS-R22 | TCTTGGTGGGTCTGTTGCTA  ATTGCCGCCGTACAAATCAA | 1199 |
| MERS-F23  MERS-R23 | AAATCATTGTCCTGCTGGCA  TGTCGAACACCTACAGCTGT | 1305 |
| MERS-F24  MERS-R24 | TGTGTGGGAAGACGGTGATT  GCAGCAAAGGAGGATAAGCC | 1308 |
| MERS-F25  MERS-R25 | ACCAGGTTTTGGAGGTGACT  AGCCCAGCAATGAAACCAAG | 1335 |
| MERS-F26  MERS-R26 | CTCCTCCTCTTCTCGGCAAT  TCCCTGAACGAGAAGCCAAT | 1495 |
| MERS-F27  MERS-R27 | CGAATCGCTTGGTTGCTACA  CCTCTACACGGGACCCATAG | 1481 |
| MERS-F28  MERS-R28 | TTCCACTGTTTTCGTGCCTG  TGCAAGTTCAATATCCGCCG | 1059 |
| MERS-F29  MERS-R29 | ACCTCAAAATGGCTGGCATG  AATGGCTCCACTGTACCGAA | 1279 |
| MERS-F30  MERS-R30 | GCACTTCTCCAGGTCCATCT  ACACTGTAGAGCTCTTCCCG | 892 |
